# Dual-Polarized Multi-Beam Fixed-Frequency Beam Scanning Leaky-Wave Antenna

**DOI:** 10.3390/s23115070

**Published:** 2023-05-25

**Authors:** Dapeng Chu, Yong Mao, Haoxiang Li, Hong Bie, Yongjin Zhou

**Affiliations:** 1 Key Laboratory of Specialty Fiber Optics and Optical Access Networks, School of Communication and Information Engineering, Shanghai University, Shanghai 200444, China; chudapeng1997@163.com (D.C.); lhxzzz@shu.edu.cn (H.L.); 2 Wuhan Marine Communication Institute, Wuhan 430205, China; 13100728680@126.com (Y.M.); 19989629966m@sina.cn (H.B.); 3 Shaanxi Key Laboratory of Artificially-Structured Functional Materials and Devices, Air Force Engineering University, Xi’an 710051, China

**Keywords:** leaky-wave antenna (LWA), surface plasmon polaritons (SPPs), dual-polarized, multi-beam, satellite communication

## Abstract

A fixed-frequency beam-scanning leaky-wave antenna (LWA) array with three switchable dual-polarized beams is proposed and experimentally demonstrated. The proposed LWA array consists of three groups of spoof surface plasmon polaritons (SPPs) LWAs with different modulation period lengths and a control circuit. Each group of SPPs LWAs can independently control the beam steering at a fixed frequency by loading varactor diodes. The proposed antenna can be configured in both multi-beam mode and single-beam mode, where the multi-beam mode with optional two or three dual-polarized beams. The beam width can be flexibly adjusted from narrow to wide by switching between multi-beam and single-beam states. The prototype of the proposed LWA array is fabricated and measured, and both simulation and experimental results show that the antenna can accomplish a fixed frequency beam scanning at an operating frequency of 3.3 to 3.8 GHz with a maximum scanning range of about 35° in multi-beam mode and about 55° in single-beam mode. It could be a promising candidate for application in the space–air–ground integrated network scenario in satellite communication and future 6G communication systems.

## 1. Introduction

The space–air–ground integrated network (SAGIN) is a crucial component of the 6G network, which is widely recognized as the future of wireless communication systems [1]. Leaky-wave antennas (LWAs) offer excellent beam scanning capabilities, along with the advantages of being low-profile, cost-effective, and low in power consumption. These characteristics make LWAs an attractive option for use as a relay terminal antenna for SAGIN applications, facilitating communication between users and satellites.

Generally speaking, LWAs are classified into two categories depending on their operating forms: uniform and periodic. Uniform LWAs are limited to forward beam scanning [2,3,4]. Periodic LWAs can complete backward-to-forward beam scanning [5,6]. However, periodic LWAs suffer from the open-stop-band (OSB) issue that the gain decreases at broadside radiation. Some methods to suppress the open-stop-band have been proposed, including loading matched stubs along the radiation direction and designing LWAs based on the balance condition of composite right/left-handed transmission line (CRLH-TL) [7,8,9,10]. To fully exploit spectrum resources, beam scanning at a fixed frequency has been successively achieved based on LWAs using multiple ways [11,12,13,14,15]. In [11], a beam-scanning LWA that can operate from 1° to 23° at 9.8 GHz was designed using a binary programmable metasurface, where a PIN diode works as a binary switch. In [12], Wang et al. proposed a sinusoidal impedance modulation-based spoof surface plasmon polaritons (SPPs) LWA loaded with varactor diodes, where a maximum scanning angle of about 45° at a fixed frequency from 5.5 to 5.8 GHz has been achieved. A triangular impedance modulation-based SPPs LWA was proposed to achieve beam scanning at fixed frequencies in the dual bands of 4 to 4.5 GHz and 5.75 to 7.25 GHz [13]. In [14], a CRLH-based LWA is proposed, whose beam scan range can reach 69° at 5 GHz. In [15], a CRLH-based LWA using ON/OFF control of PIN diode for surface impedance variation is proposed, whose beam scan angle can reach 50° at 2.45 GHz. Nevertheless, previously reported LWAs operate in a single polarization. Recently, we proposed a dual-polarized fixed-frequency beam scanning LWA, but it can only form a single beam, and the scanning angle is limited [16].

**Figure 1:**
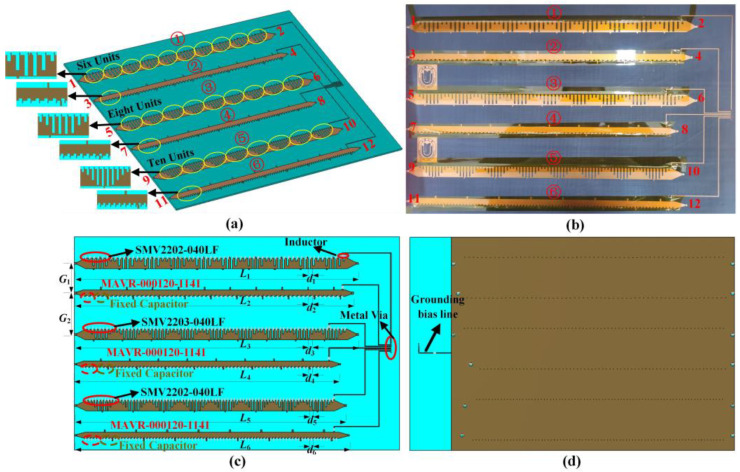
Configuration of the proposed LWA. (**a**) 3D view, (**b**) the fabricated LWA sample, (**c**) front view, (**d**) back view. (The numbers ①–⑥ correspond to the identification of each antenna in the array. The numbers 1–12 represent the port numbers used for feeding the antenna array).

In this letter, to implement dual-polarized LWAs with multi-beam capabilities for a practical mobile communication system, we propose a dual-polarized multi-beam LWA, as shown in Figure 1. The proposed LWA array consists of three groups of SPPs LWAs and a DC control circuit, each group of LWAs is loaded with varactor diodes and has different modulation period lengths. Thanks to the periodic modulation of the surface impedance, guided waves can be effectively transformed into leaky-wave radiations that possess frequency-scanning properties. Additionally, the surface impedance of the LWA can be reconfigured by adjusting the capacitance of the varactor diode through DC bias voltage, resulting in radiation beam steering across a broad angle range at a fixed frequency. Through separate and simultaneous feeding of the antenna ports, the capability to switch between narrow multi-beam and wide single-beam states is achieved. Both simulation and experimental results indicate that the antenna is capable of dual-polarized fixed-frequency beam scanning at frequencies ranging from 3.3 to 3.8 GHz, with scanning ranges of approximately 35° and 55° for multi-beam and single-beam operating states, respectively. The peak gain of the proposed antenna is 12.1 dBi, and the radiation efficiency is more than 60%. The antenna can be applied in SAGIN systems to achieve beam coverage over a large area, improving spectrum utilization and providing higher effective isotropic radiated power (EIRP) values for the service area.

## 2. Methods

Figure 1 shows the proposed dual-polarized multi-beam fixed-frequency beam scanning LWA, which is composed of six SPPs transmission lines with impedance modulation and a DC control circuit. The DC control circuit of the varactor diode is placed on the right side of the antenna array. Each parallel routing DC bias line is loaded with an inductor to separate the DC path from the RF path and avoid mutual interference. The end of the bias line is connected to the ground plane through metal vias. The entire LWA array contains three groups of dual-polarized antennas. The first group of dual-polarized antennas contains six modulation units per modulation period, the second group contains eight modulation units, and the third group contains ten modulation units. They are labeled as antennas 1st, 2nd, 3rd, 4th, 5th, and 6th from top to bottom. Antennas 1 and 5 employ the varactor diode SMV2202-040LF, with a capacitance range of 0.31 pF to 3.14 pF. Antenna 3 utilizes the varactor diode SMV2203-040LF, with a capacitance range of 0.44 pF to 4.71 pF. Their bias voltage varies from 0 to 20 V. Antennas 2, 4, and 6 utilize the varactor diode MAVR-000120-1141, with a capacitance range of 0.14 pF to 1.1 pF within a voltage variation range of 0 to 12 V. Ports 1, 3, 5, 7, 9, and 11 are feeding ports, and ports 2, 4, 6, 8, 10, and 12 are connected to matched loads to absorb the remaining electromagnetic energy. The LWA array is arranged in an X-Y-X-Y-X-Y polarization to maximize space utilization and reduce crosstalk between antennas. The antenna is designed on an F4B substrate with a thickness of 3 mm, $\varepsilon_{r}=3.5$, and tanδ=0.001.

The dimensions of each part are indicated in Table 1. Assuming that the leaky wave direction is along the transmission line, the surface impedance *Z_s_* can be described as

(1) $$Z_{s}(x)=jX_{s}[1+Mcos\left(\frac{2\pi x}{p}\right)]$$

where *X_s_* represents the average reactance of the surface, *M* represents the modulation factor, and *p* represents the modulation period.

**Figure 2:**
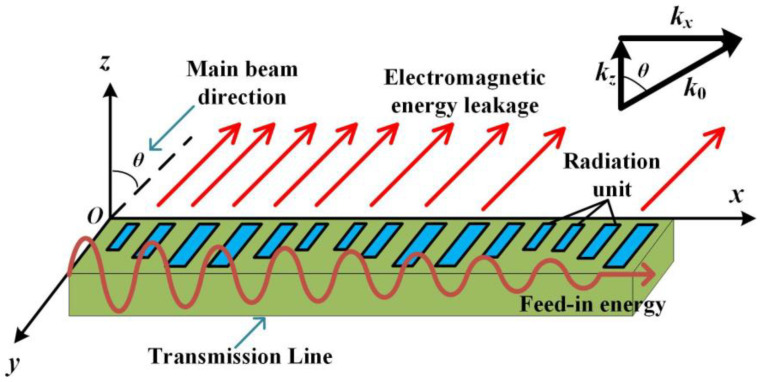
The radiation schematic of the periodic LWA.

In this design, the SPPs transmission line structure is used as the modulation unit, and the surface impedance is modulated by loading the varactor diode device and varying the notch depth to obtain the beam steering at a fixed frequency. The radiation schematic of the periodic LWA is shown in Figure 2. Unlike resonant antenna, LWA is a kind of traveling wave antenna. The electromagnetic energy will continuously leak into the free space, and the unradiated energy is absorbed by the matching load.

It has demonstrated that sinusoidal impedance surface modulation can efficiently convert non-radiative traveling waves into radiative leaky waves [17]. When *p* is determined, *M* is also determined. The fundamental mode of the SPPs antenna is slow wave mode. For −1th harmonic radiation, the main beam angle can be approximately calculated as

(2) $$\theta_{-1}=arcsin(\sqrt{1+X^{\prime 2}}-\frac{2\pi }{k_{0}p})$$

In which $X^{\prime }=X_{s}/\eta_{0}$ is the average surface reactance, $\eta_{0}$ is the free-space wave impedance, and $k_{0}$ is the free-space wave number. By changing *p*, the LWA can obtain beams with different radiation angles.

The wave number $k_{x}$ in the direction of propagation along the SPPs transmission line can be expressed as

(3) $$k_{x}=k_{0}\sqrt{1+{\left[\frac{jX_{s}(1+Mcos\left(\frac{2\pi x}{p}\right))}{\eta_{0}}\right]}^{2}}$$

Similarly, the leaky-wave antenna radiation angle θ can be described as

(4) $$\theta =arcsin\left(\frac{k_{x}}{k_{0}}\right)$$

**Figure 3:**
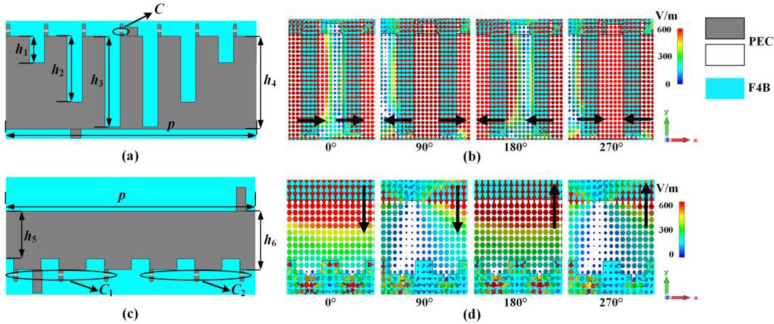
Period and electric field distribution of the dual-polarized antenna. X-polarized antenna (**a**) period, (**b**) electric field distribution; Y-polarized antenna (**c**) period, (**d**) electric field distribution. (*h*_1_ = 5.14 mm, *h*_2_ = 12.7 mm, *h*_3_ = 17.5mm, *h*_4_ = 18 mm, *h*_5_ = 9 mm, *h*_6_ = 11 mm, *p* = 43.8 mm, *C*_2_ = 1.5 pF).

To implement dual polarization, two different impedance surfaces are built on a single-sided comb structure. One is the impedance surface with different notch depths, and the other is the impedance surface with the same notch depth but using devices with different capacitance values. It is noteworthy that the electric field direction excited by the two aforementioned impedance surfaces is perpendicular to each other, which is the fundamental idea behind our design of a dual-polarized fixed-frequency beam scanning LWA. The polarization characteristics are verified by observing the electric field distribution and cross-polarization patterns. As depicted in Figure 3, it can be observed that the electric field of the LWA with different notch depths varies linearly along the *x* direction that is perpendicular to the notch direction, while the electric field of the LWA with the same notch depth varies linearly along the *y* direction that is perpendicular to the capacitance slot direction. Furthermore, Slip symmetric branching has been utilized in the antenna array to enable flexible beam modulation and weaken the OSB effect that is typically present in periodic leaky antennas.

To implement multibeam, the principle is based on Equation (1), which contains three modulation parameters, *M*, *p*, and *Zs*, respectively. The period length *p* and the modulation factor *M* are changed by varying the number of cells contained in each modulation period. By tuning the varactor, different *Zs* can be obtained at a fixed frequency. Taking the first group of antennas as an example, the X-polarized SPPs antenna has six subwavelength cells in one modulation period. The periodic modulation caused by different depths of the slot cause the radiation of high-order harmonics. The Y-polarized SPPs antenna has six subwavelength cells in one modulation period, and its radiation principle is that the periodic alternate loading of varactor diodes and fixed capacitors causes the change of surface impedance, thus causing the radiation of high-order harmonics.

**Figure 4:**
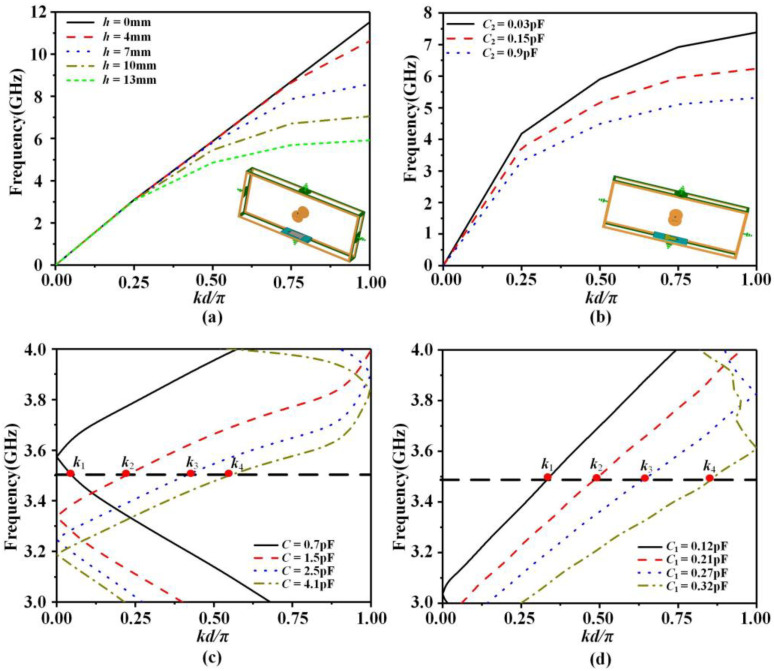
Dispersion curves of SPPs unit and modulation period. (**a**) X-polarized unit, (**b**) Y-polarized unit, (**c**) X-polarized modulation period, (**d**) Y-polarized modulation period.

The tool used for antenna simulation is CST MICROWAVE STUDIO, in which the eigenmode solver is used for the dispersion curves simulation, and the time domain solver is used for the S-parameters, gain, and radiation patterns simulation. For the unit of SPPs LWA, the most important performance is its dispersion characteristics. The dispersion curves of the X-polarized antenna unit and Y-polarized antenna unit are shown in Figure 4a,b, respectively, and it can be seen that when the slot depth of the X-polarized antenna unit increases, the cut-off frequency of the unit decreases. The depth of the slot of the Y-polarized antenna unit remains unchanged, and its dispersion curve varies with the loading capacitance. According to the dispersion curve of the antenna unit, the antenna can be designed to operate at a particular frequency. To complete the beam scanning at a fixed frequency, the wave number $k_{x}$ of propagation direction can be tuned by impedance modulation. The dispersion curves for modulation periods of the antenna are presented in Figure 4c,d. The modulation period of the X-polarized antenna has dispersion curves shown in Figure 4c. The modulation period of the Y-polarized antenna can be explained by the triangular impedance modulation evolved from the sinusoidal impedance modulation. Its dispersion curves are presented in Figure 4d. From these results, it can be seen that beam scanning at a fixed frequency can be implemented by changing the capacitance of the loaded varactor diode.

**Figure 5:**
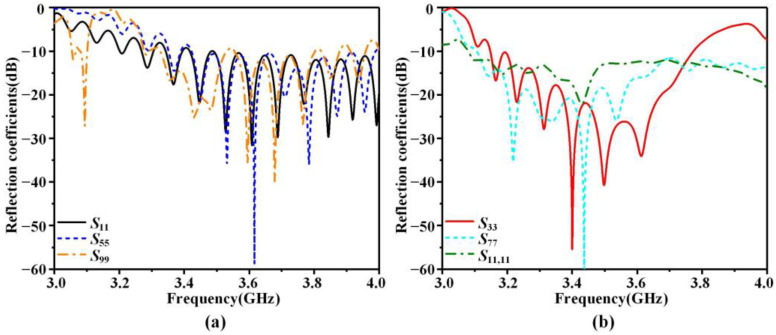
Simulated S-parameters of the LWA array. (**a**) X-polarized antenna, (**b**) Y-polarized antenna.

We have simulated the S-parameters and far-field radiation patterns of the LWA. The simulated reflection coefficients of the X-polarized and Y-polarized antennas are presented in Figure 5. It can be observed that the reflection coefficients of all six antennas in the operating frequency band of 3.3 to 3.8 GHz are consistently below −10 dB.

**Figure 6:**
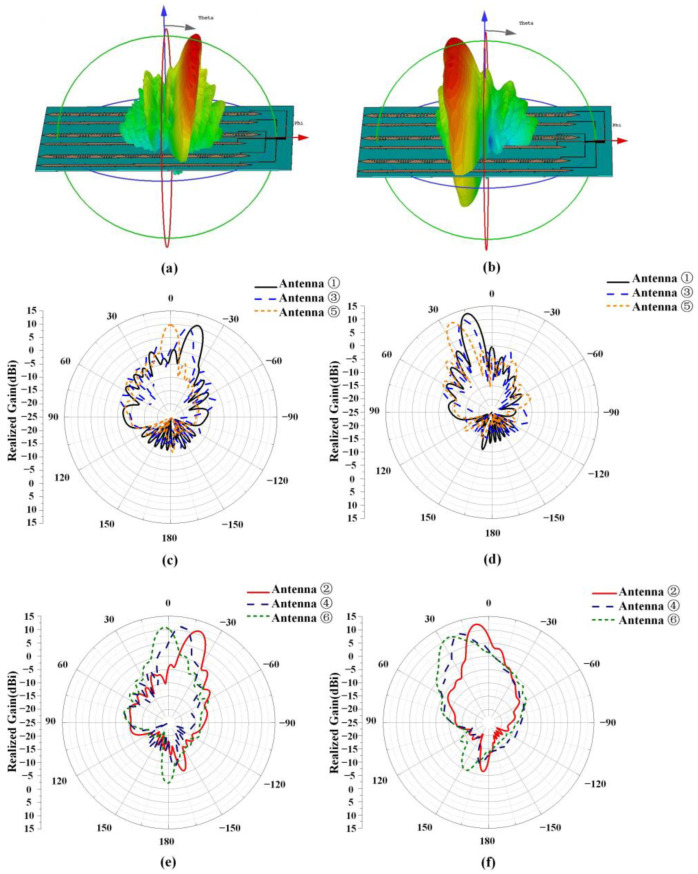
Simulated far-field radiation of the antenna at 3.5 GHz. 3-D far-field radiation patterns (**a**) backward scanning, (**b**) forward scanning; X-polarized antenna with (**c**) small capacitance state and (**d**) large capacitance state; Y-polarized antenna with (**e**) small capacitance state and (**f**) large capacitance state.

The simulated radiation patterns of the multi-beam antenna at 3.5 GHz have been presented in Figure 6, and the radiation angles of each group of x-polarized and y-polarized antennas remain identical. Figure 6a,b show the 3-D radiation patterns of the antenna. Figure 6c,d depict the radiation patterns of the X-polarized antenna with small and large capacitances, respectively. Where the large capacitance state is the capacitance of the varactor diode is 3.1 pF, the small capacitance state is the capacitance of the varactor diode is 0.5 pF. It is observed that the X-polarized antenna has a gain range of 9 to 11.2 dBi and a 3 dB beamwidth range of 8° to 11° with small capacitance, while the X-polarized antenna has a gain range of 11.3 to 13 dBi and a 3 dB beamwidth range of 8° to 10.5° with large capacitance. The maximum gain change during scanning is 2.3 dB. Similarly, Figure 6e,f illustrate the radiation patterns of Y-polarized antennas with small and large capacitances, respectively. Where the large capacitance state is the capacitance of the varactor diode is 1.1 pF, the small capacitance state is the capacitance of the varactor diode is 0.2 pF. The gain range of Y-polarized antennas under small capacitance is 11.2 to 11.6 dBi, and the 3 dB beamwidth range is 11° to 14°. Meanwhile, the gain range of X-polarized antennas under large capacitance is 10.3 to 12.4 dBi, and the 3dB beamwidth range is 13° to 18°. The maximum gain change during scanning is 1.3 dB.

## 3. Results and Discussion

**Figure 7:**
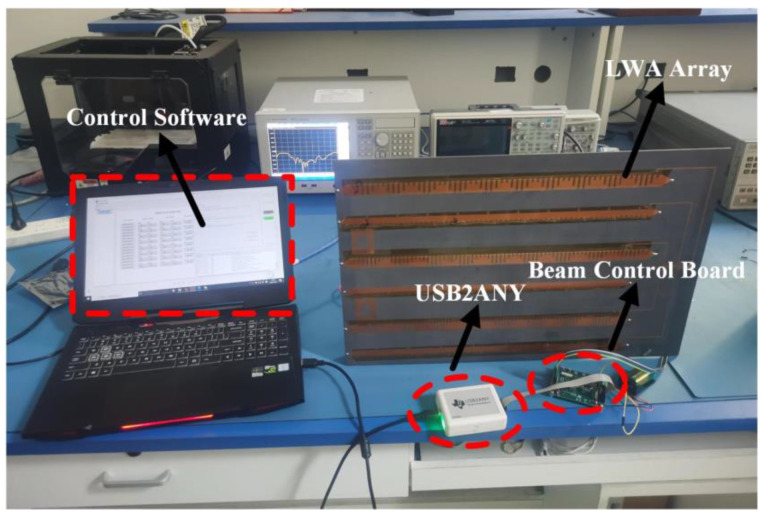
Experimental sample of the dual-polarized multi-beam scanning antenna system.

**Figure 8:**
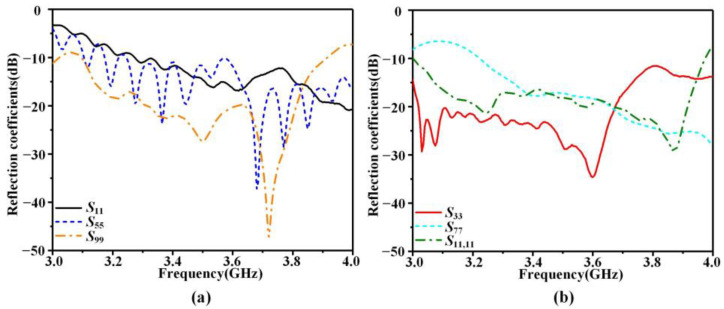
Measured S-parameters of the LWA array. (**a**) X-polarized antenna, (**b**) Y-polarized antenna.

To verify the design, the antenna system prototype shown in Figure 7 is fabricated for the actual measurement. The prototype comprises three main components: a beam control board, a USB2ANY module, and the proposed LWA array. The host computer connects to the beam control board via the USB2ANY, while the six voltage output channels of the board are connected to the metal vias of the DC bias lines of the LWA array through Dupont wires. By using specialized control software, the beam control board can be commanded to adjust the voltage loaded on both sides of the varactor diode, thereby enabling flexible beam regulation. The results are presented in Figure 8, and it can be seen that the S-parameters perform well in the range of 3.3 to 3.8 GHz. Specifically, the reflection coefficients of X-polarized and Y-polarized antennas are below −10 dB in the operating frequency band.

**Figure 9:**
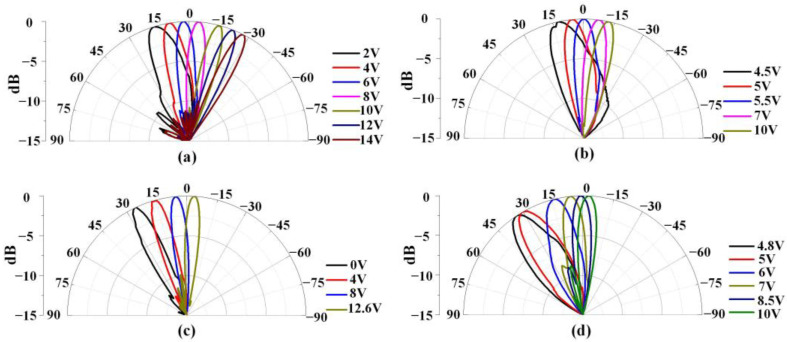
Measured normalized radiation patterns with different bias voltages loaded at 3.5 GHz. (**a**) antenna ①, (**b**) antenna ②, (**c**) antenna ③, (**d**) antenna ④.

The beam sweep performance of the antenna at a fixed frequency is indicated in Figure 9. The LWA can provide three dual-polarized beams. Antenna 1 and antenna 2 are the same set of dual-polarized fixed frequency scanning antennas with a common scanning area of −16° to 19° at 3.5 GHz. Antenna 3 and antenna 4 have a common scan area of −6° to 29° at 3.5 GHz. From the results of the measured radiation patterns, although the maximum beam scan range of antenna 1 in the multi-beam state can cover −28° to 19°, considering the comprehensive dual-polarized performance, each group of dual-polarized antenna common beam scan range is about 35°. Taking antenna 3 as an example to analyze the test results, when the bias voltage range varies from 0 V to 12.6 V, the capacitance of the varactor diode changes from 4.7 pF to 0.6 pF, and the antenna scans from 29° to −4°. The side beam level of the antenna is more than 10 dB lower than the main beam level during the entire scan. The differences between the measurement results and the simulation results may be due to the different parasitic parameters generated by the varactor diode when high and low voltages are applied, which cannot be accurately simulated by the simulation software.

**Figure 10:**
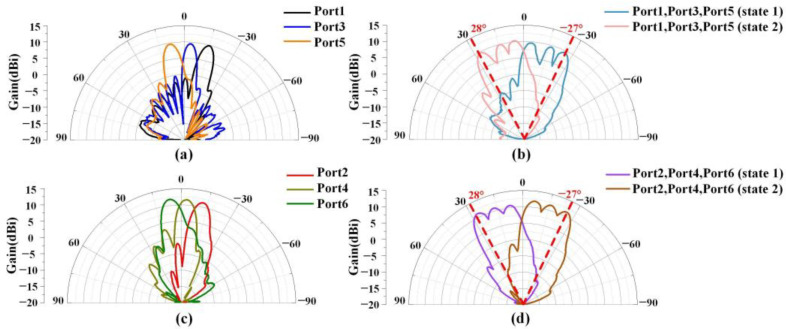
Measured radiation patterns for excitation of different feeding ports. X-polarized antenna in (**a**) multi-beam operating state, (**b**) single-beam operating state; Y-polarized antenna (**c**) multi-beam operating state, (**d**) single-beam operating state.

Next, we will investigate the single-beam mode and the multi-beam mode. The measured radiation patterns are displayed in Figure 10 when the different feeding ports of the antenna array are excited. The antenna array can produce multiple directional radiation beams when fed individually from different ports, which is a multi-beam operation. The X-polarized antenna gain ranges from 9.7 to 9.9 dBi with a 3 dB beam width of about 9°, while the Y-polarized antenna gain ranges from 11.3 to 11.7 dBi with a 3 dB beam width of about 11°. When multiple ports are fed simultaneously, the antenna array can generate a wider beam, which is the single-beam working state. The X-polarized antennas have a beam width of about 31°, and the Y-polarized antennas have a beam width of about 32° in the single-beam working state. The scanning range of the single beam can be flexibly adjusted by varying the voltage value of the varactor diode on the antenna, allowing for a total scanning range of approximately 55°, with the ability to scan from −27° to 28°.

**Figure 11:**
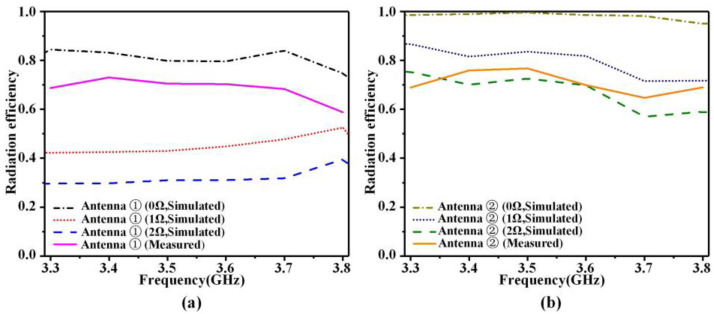
Radiation efficiency of the dual-polarized antenna. (**a**) X-polarized antenna, (**b**) Y-polarized antenna.

Because the parasitic resistance of the varactor diode has a large impact on the radiation efficiency of the antenna, so we take the first group of the dual-polarized antenna as an example and change the parasitic resistance value to observe the corresponding variation in radiation efficiency; the results are displayed in Figure 11. Without considering the effect of device parasitic resistance, the radiation efficiency of the dual-polarized antenna is higher than 75%. The parasitic resistance of the device will cause the radiation efficiency of the active antenna to decrease. It can be seen that Y-polarized antennas are less affected by the parasitic resistance because the number of loaded varactor diodes is less, so when the parasitic resistance of the device is 2 Ω, the radiation efficiency of more than 56% can still be achieved in the operating frequency band. However, the X-polarized antennas suffer from a higher Ohmic loss during simulation, which is because the X-polarized antenna uses a larger number of active devices. The measurement results show that the actual radiation efficiency of the dual-polarized antenna exceeds 60% in the frequency range of 3.3 to 3.8 GHz.

Table 2 provides a comparison of performances between the proposed dual-polarized multi-beam fixed-frequency beam scanning LWA and the previously reported LWAs. In Table 2, $\lambda_{0}$ is the wavelength at the center frequency of the working band for LWAs. Although these previous works have achieved fixed-frequency beam scanning, most of them are single-polarized and can only form a single beam. The proposed antenna is dual-polarized and can form multiple beams, while the beam width can be flexibly adjusted by switching between multi-beam and single-beam states. The proposed antenna also performs well in terms of gain and beam scanning range.

## 4. Conclusions

An SPPs LWA array with dual-polarized, switchable, and steerable multi-beam at fixed frequency is proposed. In the frequency range of 3.3–3.8 GHz, this antenna has the capability to modify the voltage loaded on the varactor diode to achieve a maximum scanning range of 35° for multi-beams and 55° for single-beams. Additionally, the beam width is adjustable by switching between the multi-beam and single-beam modes. The peak gain of this antenna is 12.1 dBi, while the radiation efficiency exceeds 60%. Given these capabilities, this antenna holds great potential for use as a relay antenna in future satellite communication within the SAGIN. The antenna’s measured results exhibit excellent agreement with the simulated results, validating its superior performance.

## Figures and Tables

**Table 1:** The optimized dimensions of the proposed antenna.

Parameter	Values (mm)	Parameter	Values (mm)
*L*_1_	480	*d*_1_	7.3
*L*_2_	472	*d*_2_	7.3
*L*_3_	480	*d*_3_	6.1
*L*_4_	450	*d*_4_	6.1
*L*_5_	460	*d*_5_	5.2
*L*_6_	465	*d*_6_	5.3
*G*_1_	50	*G*_2_	70

**Table 2:** Comparison of the proposed LWA with the reported references.

Ref.	Freq. (GHz)	Length (λ_0_)	Polarization	Beam Width Tuning	Scanning Mode	Beam Scanning Range	Maximum Gain (dBi)
[12]	5.5–5.8	6.2	Single	No	Single-beam	45°	8
[13]	4–4.5 and 5.75–7.25	4.65 and 7.15	Single	No	Single-beam	80° and 22°	13.8
[14]	4.75–5.25	2.6	Single	No	Single-beam	69°	6.4
[15]	2.45	2.29	Single	Yes	Single-beam	50°	8
[16]	3.4–3.7	5.4	Dual	No	Single-beam	39°	9.7
This work	3.3–3.8	5.6	Dual	Yes	Single-beam and Multi-beam	55° and 35°	12.1

## Data Availability

Not applicable.

## References

[1] Kuang L., Chen X., Jiang C., Zhang H., Wu S. (2017). Radio resource management in future terrestrial-satellite communication networks. IEEE Wirel. Commun..

[2] Liu J., Jackson D.R., Long Y. (2012). Substrate integrated waveguide (SIW) leaky-wave antenna with transverse slots. IEEE Trans. Antennas Propag..

[3] Yang S., Ling H. (2013). Application of a microstrip leaky wave antenna for range–azimuth tracking of humans. IEEE Geosci. Remote Sens. Lett..

[4] Mallahzadeh A., Mohammad-Ali-Nezhad S. (2014). Long slot ridged SIW leaky wave antenna design using transverse equivalent technique. IEEE Trans. Antennas Propag..

[5] Karmokar D.K., Esselle K.P. (2015). Periodic U-slot-loaded dual-band half-width microstrip leaky-wave antennas for forward and backward beam scanning. IEEE Trans. Antennas Propag..

[6] Shi S., Qian Z., Cao W. (2019). SIW cavity-backed self-diplexing leaky-wave antenna. Electron. Lett..

[7] Lyu Y., Meng F., Yang G., Wang P., Wu Q., Wu K. (2018). Periodic leaky-wave antenna based on complementary pair of radiation elements. IEEE Trans. Antennas Propag..

[8] Dong Y., Itoh T. (2011). Composite right/left-handed substrate integrated waveguide and half mode substrate integrated waveguide leaky-wave structures. IEEE Trans. Antennas Propag..

[9] Dong Y., Itoh T. (2012). Substrate integrated composite right-/left-handed leaky-wave structure for polarization-flexible antenna application. IEEE Trans. Antennas Propag..

[10] Karmokar D., Chen S.-L., Bird T., Guo Y.J. (2019). Single-layer multi-via loaded CRLH leaky-wave antennas for wide-angle beam scanning with consistent gain. IEEE Antennas Wirel. Propag. Lett..

[11] Wan X., Chen T.Y., Chen X.Q., Zhang L., Cui T.J. (2018). Beam forming of leaky waves at fixed frequency using binary programmable metasurface. IEEE Trans. Antennas Propag..

[12] Wang M., Ma H.F., Zhang H.C., Tang W.X., Zhang X.R., Cui T.J. (2018). Frequency-fixed beam-scanning leaky-wave antenna using electronically controllable corrugated microstrip line. IEEE Trans. Antennas Propag..

[13] Wang M., Ma H.F., Tang W.X., Zhang H.C., Jiang W.X., Cui T.J. (2019). A dual-band electronic-scanning leaky-wave antenna based on a corrugated microstrip line. IEEE Trans. Antennas Propag..

[14] Chen S., Karmokar D.K., Li Z., Qin P., Ziolkowski R.W., Guo Y.J. (2019). Continuous beam scanning at a fixed frequency with a composite right-/left-handed leaky-wave antenna operating over a wide frequency band. IEEE Trans. Antennas Propag..

[15] Shaw R., Mandal M.K. (2020). Broadside scanning fixed frequency LWA with simultaneous electronic control of beam angle and beamwidth. IEEE Trans. Antennas Propag..

[16] Li H.X., Zhou Y.J. (2021). Dual-polarized fixed-frequency beam scanning leaky-wave antenna for 5G communication. Appl. Comput. Electromagn. Soc. J..

[17] Oliner A., Hessel A. (1959). Guided waves on sinusoidally-modulated reactance surfaces. IRE Trans. Antennas Propag..

